# Effect of ethnic origin and newborn gender on mother–infant bonding

**DOI:** 10.1007/s00404-025-08003-9

**Published:** 2025-03-28

**Authors:** Hagar Brami, Eyal Sheiner, Tamar Wainstock, Talya Lanxner Battat, Inbal Reuveni, Tom Leibson, Gali Pariente

**Affiliations:** 1 Department of Obstetrics and Gynecology, Soroka University Medical Center, Ben Gurion University of the Negev, POB 151, Beer-Sheva, Israel; 2 Department of Public Health, Faculty of Health Sciences, Ben-Gurion University of the Negev, Beer-Sheva, Israel; 3 Psychiatric Division, Sourasky Medical Center, Tel Aviv-Yafo, Israel; 4 Department of Pediatrics, Mount Sinai Hospital, Toronto, ON Canada; 5 Division of Clinical Pharmacology and Toxicology, Hospital for Sick Children, Toronto, ON Canada

**Keywords:** Mother-infant bonding, Gender, Ethnicity

## Abstract

**Background:**

Fetal gender has been shown to influence pregnancy and perinatal outcomes. Adequate mother–infant bonding may have a positive effect on maternal and child's mental health further reducing the risk for maternal depressive symptoms and child's psychopathology.

**Objective:**

We aimed to assess the association between newborn gender and mother–infant bonding in the Arab Bedouin women in comparison to the Jewish population residing in the same area.

**Methods:**

A cross-sectional study was performed on women who delivered singletons during bonding questionnaire (PBQ). A second analysis of the four sub-scales of the PBQ questionnaire was conducted. The risk for post-partum depression was assessed using the Edinburgh Postnatal Depression Scale (EPDS) questionnaire. Self-reported questionnaires were administered to obtain sociodemographic data and additional information was drawn from women’s medical records. Multivariable linear regression models were constructed to control for potential confounders.

**Results:**

The final analysis included 218 women, of whom 98 (44.9%) were of Bedouin origin and 120 (55.1%) were of Jewish origin. While total PBQ score was significantly lower among Bedouin women delivering male infants compared to female infants, demonstrating better mother–infant bonding (8.8 ± 3.8 vs. 15.1 ± 9.5, *p* = 0.010), no difference was found in the total PBQ score between Jewish women delivering male or female infants. Multivariable linear regression models controlling for maternal age, primiparity, and EPDS score demonstrated better mother–infant bonding when delivering a male infant compared to female infant among the Bedouin women (Beta 5.86, 95% CI 1.80–9.90, *p* = 0.006). Among Jewish women, no independent association was found between infant gender and mother–infant bonding.

**Conclusion:**

Better mother–infant bonding was found among Bedouin women delivering male infants compared to females. Modernization is challenging the Bedouins’ patriarchal society; therefore, attempts to strengthen mother–infant bonding of the female gender are to be considered.

## What does this study add to the clinical work

**Table:** 

Cultural values can significantly shape postpartum experiences and mother-infant relationships.	

## Introduction

Depression during pregnancy and the post-partum period affects 15–20% of women, with a higher prevalence in traditional societies and those of low socioeconomic status [1].Research on perinatal depression has focused mainly on universal factors. However, cultural differences play a pivotal part in transition to motherhood and with regard to emotional difficulties around pregnancy and childbirth [2].

The emotional state of the mother toward the infant, defined as mother–infant bonding, is the basis for the child's social-emotional development [3]. Mother–infant bonding is believed to begin at pregnancy and continues into early childhood [4]. Adequate mother–infant bonding can have a positive effect on maternal mental health by reducing the risk for depressive symptoms [5]. Disturbance in mother–infant bonding has adverse implications on both the mother and child, including anxiety, aggression, resentment, and rejection of the child [6]. Impaired mother–infant bonding is associated with maternal post-partum depression [7], higher risk for maltreatment, neglect, and child abuse in the future [8, 9]. As for the children, impaired mother–infant bonding has been associated with a negative effect on child cognitive development, and a higher risk for psychopathology later in life [10–12].

Fetal gender has been shown to influence pregnancy and perinatal outcome [13–16]. Male fetus pregnancies are at higher risk for gestational diabetes mellitus, cord incidents, fetal distress, and low Apgar scores. Higher rates of labor dystocia, preterm labor, and cesarean section have also been demonstrated in male fetus pregnancies [17, 18]. The biological mechanism of gender difference has not been fully discovered yet, and studies suggest hormonal, physiological, and genetic factors play a major role in gender differences [19].

The term “son preference” is used to describe the importance and value of having a male in many societies in Eastern and Southern Asia [20]. Male preference may have several explanations such as: patriarchal society, religion, economic burden for dowry payment, financial security for the older parents, and support in the form of physical labor [21]. A higher value for the male gender over the female gender was found in China, Turkey, and India with a great impact on post-partum depression [2].

Two populations reside in the southern district of Israel: the Jewish population and the Bedouin population. The Bedouins in Israel are a unique nomadic Muslim Arab minority with cultural, historical, and distinct social codes. For the most part, the Bedouin society is conservative and maintains its traditional values and customs [22]. The typical Bedouin family is large, patriarchal, and characterized by a high fertility rate compared to the Jewish population [4]. As in a patriarchal society, the status of men is considered to be higher than the status of women [23]. The process of modernization within the society has led to a situation in which a significant part of the Bedouin sector is considered sociologically as a "population in transit" [22]. Bedouin women suffer from high rates of post-partum depression ranging from 26 to 43% [24]. Polygamous marriage and consanguinity marriage are associated with higher rates of post-partum depression in the Bedouin society [25]. The compulsory national health insurance system implemented in Israel in 1995 provides health care to the entire population through nonprofit health maintenance organizations. Like all other Israeli citizens, regardless of cultural and religious differences, the Bedouin community is covered by the same national health insurance system.

So far, most research on mother–infant bonding and post-partum depression was conducted within the Western population [26] using research tools that were mostly designed for English-speaking participants. Our study design has allowed us to recruit both Jewish and Bedouin participants, who were able to share with us some of their thoughts and emotions through a validated questionnaire printed in the woman’s first language. Considering the sociodemographic transition, which the Bedouin population is undergoing, our aim in this study was to investigate the potential connections between newborn’s gender and the reported mother–infant bonding.

Our hypothesis is that patterns of mother–infant bonding with respect to the newborn’s gender would differ between Jewish and Bedouin women. Specifically, we hypothesize that Bedouin mothers would report a stronger bonding with their male newborns when compared to Jewish women, and that post-partum depression rates among Bedouin women may be significantly affected by their child’s gender.

## Methods

### Settings and population

The study took place in Soroka University Medical Center (SUMC), the sole medical center in the southern part of the country that provides health services to both the Jewish and Bedouin populations. Being the only medical center in the area, SUMC constitutes the largest birthing center in the country with more than 17,000 births a year. Although the Jewish population who lives in southern Israel outnumbers the Bedouin minority, more than half of all births in SUMC are attributed to the Bedouin community. The study included women who delivered singleton live births between March and May 2020 at SUMC.

To be included in the study, women had to be at least 18 years old at the time of assessment and to provide informed consent to answer the study questionnaires. Women who had a cesarean delivery, newborns with congenital anomalies and chromosomal abnormalities, and newborns admitted to the neonatal intensive care unit (NICU) were excluded from the study. Women with past or present psychotic symptoms, suicidality, or substance abuse according to medical health records were also excluded from the study.

The study was approved by the local Institutional Review Board (IRB approval # 0079-20-SOR).

### Study design

A cross-sectional study was performed in women during the immediate post-partum period (first 3 days after delivery), focusing on healthy women hospitalized in the maternity wards of the SUMC. During their admission after labor, suitable women were identified through an initial review of medical records. Women who provided consent to participate in the study answered the study questionnaires. Each woman was evaluated at a single time point. The study involved answering a questionnaire regarding mother–infant bonding. The questionnaire results were compared between mothers to male and female neonates in both populations.

### Post-partum mother–infant bonding assessment

The clinical diagnosis of mother–infant bonding was assessed by the Post-partum Bonding Questionnaire (PBQ) [27]. PBQ is a valid screening tool for mother–infant bonding disorders [28]. PBQ consists of 25 statements, rated on a 6-point Likert scale (from “always” to “never”—0 to 5 points each) assessing the mother’s feelings and attitudes toward her baby. The maximum total score is 125, while a higher score indicates impaired bonding. According to Brockington et al. [27], we performed a factor analysis and four sub-scales were recognized: impaired bonding (12 items), rejection and anger (7 items), anxiety about care (4 items), and risk of abuse (2 items). The sum of scores for each sub-scale was calculated and an unfavorable parent–child relationship was defined if the rating showed a score of 12 or more on the first factor, 17 or more on the second, 10 or more on the third, and 3 or more on the fourth. With this cutoff level, Brockington et al [29]. found specificity for ‘‘normal’’ mothers between 0.85 and 1.00 for the four sub-scales. The sensitivity varies between the sub-scales, but the sub-scale impaired bonding seemed to identify over 90% of mothers with some form of bonding disorders. The questionnaire has been translated into Hebrew and Arabic for this study using a back-translation technique.

### Post-partum depression risk assessment

Post-partum depression risk was measured using the Hebrew and Arabic version of the EPDS questionnaire. The American College of Obstetricians and Gynecologists has recommended using a standardized questionnaire, such as the EPDS, as a screening tool to improve the identification of women with post-partum depression [30]. The EPDS is composed of ten items specially designed to screen post-partum women for depressive symptoms [31]. The instrument has established validity and reliability [32]. A cutoff of ≥ 13 has been applied to identify participants at high risk for depressive symptoms. A score ≥ 10 (maximum score is 30) has a sensitivity of 0.83 (95% confidence interval [CI] 0.81–0.86) and specificity of 0.85 (95% CI 0.84–0.86) for minor and major depression.

### Sociodemographic control variables

Self-report questionnaires were completed including sociodemographic data such as maternal age, educational status, and marital status. Patients’ medical records were reviewed for additional information, including clinical maternal characteristics such as gravidity and parity, history of mental illness, and data regarding pregnancy complications such as hypertensive disorders, gestational diabetes, and post-partum hemorrhage.

### Statistical analysis

Statistical analysis was performed using SPSS version 26.0. Comparison of continuous variables was performed using Student’s* t* test and Chi-square test was used to examine differences in the distribution of categorical variables. Multivariable linear regression models were constructed to examine the association between the independent and dependent variables while adjusting for confounding. The strategy for the model building was as follows: background characteristics were compared between the study groups. Variables associated with the exposure (i.e., were different between the study groups) were suspected as confounding variables and tested in the multivariable models, to determine whether they were also associated with the outcome variable, and therefore possibly confounding the association between the exposure and the outcome. Interactions between the independent variables were examined. No interactions between the independent variables and exposure were found.

## Results

The final analysis included 218 women, of whom 98 (44.9%) were of Bedouin origin and 120 (55.1%) were of Jewish origin.

Maternal, clinical, and demographic characteristics were divided by infant gender and are presented in Table 1. Bedouin women who delivered male infants were older and had higher rates of history of psychiatric disorder compared to Bedouin women who delivered female infants (14.6% vs 2.0%, *p* = 0.023). No significant differences were demonstrated between the groups regarding marital and educational status, parity, pregnancy complications, and lactation. Table 1 shows no differences in maternal demographic and clinical characteristics between Jewish women who deliver male infants compared to female infants.

**Table 1:** Maternal clinical and demographic features, pregnancy, and delivery data in Bedouin and Jewish women divided by newborn gender

Characteristics	Bedouin women			Jewish women		
	Male (*n* = 48)%	Female (*n* = 50)%	*p* value	Male (*n* = 48)%	Female (*n* = 50)%	*p* value
Maternal age, years (mean ± SD)	27.6 ± 5.0	25.5 ± 4.5	0.034	30.9 ± 4.8	31.1 ± 5.1	0.848
Marital status						
Married	100%	95.8%	0.162	93.9%	91.5%	0.617
Other	–	4.2%		6.1%	8.5%	
Educational status						
Elementary school	6.1%	7.1%	0.634	–	–	0.623
High school	69.7%	57.1%		36.7%	29.4%	
University- first degree	15.2%	21.4%		26.5%	23.5%	
University- second degree and above	6.1%	14.3%		36.7%	47.1%	
History of psychiatric illness	14.6%	2.0%	0.023	12.9%	16.0%	0.626
Gravidity						
1	12.5%	24.0%	0.337	12.9%	14.0%	0.984
2–4	54.2%	46.0%		62.9%	62.0%	
≥ 5	33.3%	30.0%		24.3%	24.0%	
Parity						
1	14.6%	24.0%	0.238	18.6%	24%	0.470
≥ 2	85.4%	76.0%		81.4%	76%	
Fertility treatments	6.3%	2.0%	0.288	5.7%	2.0%	0.315
Preterm delivery	18.8%	12.0%	0.354	18.6%	14.0%	0.508
Pregnancy complications*	4.2%	2.0%	0.534	2.9%	8.0%	0.203
Low birth weight	4.2%	4.0%	0.967	7.1%	4.0%	0.469
Gestational age at birth, weeks (mean ± SD)	38.8 ± 1.5	39.2 ± 1.4	0.158	38.2 ± 4.9	38.5 ± 5.7	0.771
Lactation						
Exclusive breastfeeding	32.4%	27.6%	0.917	53.2%	57.6%	0.325
Supplementary formula- feeding	64.7%	69%		25.5%	33.3%	
Only formula- feeding	2.9%	3.4%		21.3%	9.1%	

*Pregnancy complications include gestational diabetes mellitus, hypertensive disorders, and post-partum hemorrhage

EPDS results divided by infant gender are presented in Table 2. While Bedouin women who delivered male infants had a lower risk for post-partum depression, Jewish women demonstrated the opposite: a lower risk for depression when delivering female infants. Yet, these differences were not statistically significant in both groups.

**Table 2:** Edinburgh Postnatal Depression Scale (EPDS) results among Bedouin and Jewish women divided by newborn gender

	Bedouin women			Jewish women		
	Male (*n* = 48)%	Female (*n* = 50)%	*p* value	Male (*n* = 48)%	Female (*n* = 50)%	*p* value
Total EPDS score ≥ 10	16.7%	24.5%	0.341	17.6%	6.1%	0.066
Total EPDS score ≥ 13	6.3%	18.4%	0.070	10.3%	4.1%	0.213
Suicidal ideations (according to question number 10 in EPDS questionnaire)	2.1%	2.0%	0.977	1.5%	2.0%	0.826

Mother–infant bonding assessed by PBQ is described in Table 3. While the total PBQ score was significantly lower among Bedouin women delivering male infants, demonstrating better mother–infant bonding (8.8 vs. 15.1, *p* = 0.010), no difference was found in the total PBQ score between Jewish women delivering male or female infants (7.8 vs. 7.3, *p* = 0.712). Likewise, while impaired bonding sub-scale score was significantly lower in Bedouin women who delivered males, indicating better mother–infant bonding (6.8 vs. 10.0, *p* = 0.015), no difference in impaired bonding sub-scale score was shown between genders in Jewish women. In both groups, no women showed rejection and anger, or anxiety about care.

**Table 3:** Post-partum Bonding Questionnaire (PBQ) results in Bedouin and Jewish women with male newborn and with female newborn (mean scores ± SD)

Post-partum bonding questionnaire (PBQ) results (mean scores ± SD)	Bedouin women			Jewish women		
	Male (*n* = 35) mean (SD)	Female (*n* = 35) mean (SD)	*p* value	Male (*n* = 35) mean (SD)	Female (*n* = 35) mean (SD)	*p* value
Nominal scores						
Impaired bonding (*12 items*)	6.8 (3.0)	10.0 (5.2)	0.015	4.1 (3.5)	3.5 (3.5)	0.512
Rejection and anger (*7: items*)	1.1 (1.9)	2.0 (2.6)	0.087	1.3 (2.0)	1.2 (1.9)	0.753
Anxiety about care (*4: items*)	1.1 (1.8)	2.0 (2.3)	0.057	2.2 (2.1)	2.5 (2.5)	0.521
Risk of abuse (*2: items*)	–	–	NA	0.1 (0.6)	0.1 (0.1)	0.239
Total PBQ score	8.8 (3.8)	15.1 (9.5)	0.010	7.8 (6.8)	7.3 (6.6)	0.712

Multivariable linear regression models controlling for maternal age, primiparity, and EPDS score were constructed to study the association between infant gender and mother–infant bonding among Bedouin and Jewish (Table 4) women. Among Bedouin women, an independent association was demonstrated between infant gender and mother–infant bonding, demonstrating better mother–infant bonding when delivering a male infant compared to a female infant (beta 5.86, 95% CI 1.80–9.90, *p* = 0.006, model no.1, Table 4). Among Jewish women, no independent association was found between infant gender and mother–infant bonding (beta 0.90, 95% CI − 1.21–3.01, *p* = 0.402, model no.1, Table 4). Similar results were demonstrated using two additional multivariable regression models in Bedouin and Jewish women, showing an independent association between infant gender and impaired bonding sub-scale score among Bedouin women and no association among Jewish women (Beta 3.2, 95% CI 0.66–5.75, *p* = 0.015 and beta 0.1, 95% CI − 0.99–1.31, *p* = 0.783 for Bedouin and Jewish women, respectively, model no. 2, Table 4).

**Table 4:** Multivariable linear regression models

	Bedouin women			Jewish women		
	Beta	95% CI	p value	Beta	95% CI	*p* value
Model no.1: male gender and mother–infant bonding total score						
Male gender (versus female gender)	5.86	1.80, 9.90	0.006	0.90	− 1.21, 3.01	0.402
Maternal age (years)	0.12	0.55, − 0.29	0.550	− 0.04	− 0.27, 0.18	0.678
Primiparity	− 3.11	− 8.65, 2.40	0.261	3.00	0.28, 5.72	0.031
EPDS > 10	9.19	3.37, 15.01	0.003	9.19	8.90, 15.12	> 0.001
Model no.2: male gender and *impaired* mother–infant bonding sub-scale score						
Male gender (versus female gender)	3.2	0.66–5.75	0.015	0.1	− 0.99–1.31	0.783
Maternal age (years)	− 0.2	− 0.27–0.22	0.831	0.03	− 0.08–0.15	0.586
Primiparity	− 2.1	− 5.53–1.24	0.208	1.6	0.18–3.13	0.027
EPDS > 10	3.0	− 0.61–6.66	0.100	5.4	3.71–7.14	N/A

The association between male gender and mother–infant bonding and the association between male gender and *impaired* mother–infant bonding in Bedouin and Jewish women

## Discussion

In this study, in line with our first hypothesis, we interrogated the relationship between ethnic origin and infant gender on mother–infant bonding. Bedouin women who delivered male as compared to female infants demonstrated better mother–infant bonding measured by the PBQ. No independent association was found between infant gender and mother–infant bonding among Jewish women. EPDS results for Bedouin women have shown a trend toward lower risk for post-partum depression following male infant delivery, although this trend was not statistically significant.

### Infant gender and post-partum depression

Since the book “Maternal–infant bonding'' was written by Klaus and Kennell in 1976 [33], many studies have investigated factors that influence bonding between mothers and their children [34]. Nonetheless, little is known about the association between infant gender and mother–infant bonding.

Infant gender poses a cultural dispute. The fetal gender has a major impact on the mother and the family [35]. Cultures with strong son preference in Asia, Africa, Eastern Europe, and the Caucasus region suffer from male-biased sex ratios in the population, partly due to sex-selective terminations of pregnancies [21].Culture plays a dominant role in predicting mother–infant bonding and post-partum depression [36],with higher rates among immigrants and ethnic minorities in the same population [37],as well as deliveries during wartime [38].While most studies on risk factors for mother–infant bonding and post-partum depression were done in Western societies, a study within a Middle Eastern population found a strong effect on the history of depression and high social support [26],as well as emotion regulation impacted by pain during the immediate post-partum period [39], emphasizing the place of tradition and cultural norms on mother–infant bonding.

The risk factors for post-partum depression share similar themes cross-culturally, with one notable exception; the impact that the gender of the infant had on post-partum depression. This impact of offspring gender was reported in the literature from India [1], Turkey [40], and China [41]. Studies describing a transcultural analysis of post-partum depression, demonstrating lower risk for post-partum depression among women delivering male infants, support this finding [2]. Son preference is a characteristic of a patrilineal family culture, where the son stays with his family after marriage and takes care of his parents, while daughters leave after marriage for their husbands' households [42]. Possible theories for son preference rely on an earlier conception that the female gender is viewed as an economic burden to the family including dowry payments and eventually, after marriage, leaving to her husband’s family [43], while families that live in rural areas need males for physical work [44]. In China, the policy of having one child contributed to male preferences, as males can help take care of their parents in elderliness and continue the family’s business and dynasty [45]. Nowadays, the “two children policy” has not changed much. If the first baby is a girl, the family strongly desires for the second to be a boy. The government is against selective induced abortion, so antenatal examination of the fetus's gender is forbidden by law [46]. In India, sex-selective abortions are banned by law, but not enforced due to the legality of abortions [47]. Concordant to our study, a Turkish study found male gender to correlate with better mother–infant bonding assessed at 2 months of age [48]. Despite many years of modernization and Westernization, the delivery of a female infant in Hong Kong, was demonstrated to be unfavored, showing that preferences regarding infant gender are related to social-cultural values rather than a biological event [45]. A multinational analysis including 24 European countries aimed to assess the effect of the preferred gender composition of offspring, for both men and women, and whether this preferred gender composition affects the parental intent to have a third child. Using the European Social Survey, the authors demonstrated the presence of a mixed-sex preference. In countries with a high risk of poverty in old age, a preference [45] for sons was found, particularly in men [49].

A German study including 862 mothers aimed to investigate the association between impaired bonding and post-partum depression by using EPDS and PBQ questionnaires. They found that a higher EPDS score (higher risk for post-partum depression) had a strong correlation with a higher PBQ score (impaired bonding), thus showing impaired bonding is well associated with post-partum depression [50]. Another study from Sweden investigated the influence of paternal and maternal depressive symptoms on impaired bonding. The study used EPDS and PBQ questionnaires in 727 couples for the evaluation of maternal and paternal depressive symptoms as a primary aim, and a self-report questionnaire to assess the influence of marital problems on bonding as a secondary aim. Impaired bonding was found in high prevalence in couples suffering from depressive symptoms and was also influenced by marital problems [51]. An Israeli study of 1128 participants compared post-partum depression rates between Jewish and Arab women (853 Jewish and 275 Arab). Post-partum depression rates were 20.8% and 7% for Arab and Jewish women, respectively. The differences were mostly due to ethnic inequalities and the notion that minorities tend to suffer more from post-partum depression [52]. Similar results were found in a research study during the COVID-19 pandemic with higher perinatal depression rates among Arab women [53]. A study on different ethnic groups in New York strengthened the relation between being a minority/ethnic disparity and higher rates of post-partum depression [54].In our study, Bedouin women who delivered male infants had a non-significant trend toward lower risk for post-partum depression. Possible explanations for our finding are that the bonding impairment associated with delivering a female infant that was found among Bedouin mothers resulted from other reasons rather than the risk for post-partum depression.

In the present study, delivery of male infants in Bedouin women was associated with better mother–infant bonding. Our results correlate with the concept of son preference, which characterizes patriarchal cultures. The Bedouin society has patriarchal features and, although not as common as in the past, polygamy is still accepted [55] with great influence of male gender and age on the family rules and norms [23]. Over the past half-century, the Bedouins have been influenced by the process of modernization, even in rural areas; while 60% of the Bedouin society have moved to live in planned cities abandoning agricultural work, 40% live in rural villages and partly maintain the traditional way of living [22]. As in a patriarchal society, some of the Bedouin women may be underprivileged [56] and as some marriages are pre-arranged at a young age, some women are subordinate to their husbands [57]. Women are expected to bear children and are treated with honor when they do so, and large families are a sign of the tribe’s power [58] especially when delivery of male infants occurs [56]. Traditionally grazing sheep and handicrafts were solely done by the women who played a dominant role in the house’s economy. Today, even though the reality has changed, women in patriarchal societies are somewhat expected to stay at home [59]. Nevertheless, nowadays, there is a process of Westernization among Bedouin women, with increasing incidence of higher education, focusing on careers, and carrying a tremendous change in the hierarchy [60]. The perception of modernization and women’s status is changing in many cultures in the developed countries [61–63]. Feminist women stand against patriarchy [58], and fight for breaking boundaries for women [64, 65].

### Strengths and limitation

There are several strengths to the study. First, this study presents a relatively large cohort of two different ethnic groups of women, residing in the same area, sharing the same health facilities and medical center which is the sole birthing center in the southern part of the country. Health care is provided to the entire population through nonprofit health maintenance organizations and through the hospital. Both the Bedouin and the Jewish communities are covered by the same national health insurance system. This allows parsing apart the cultural differences from socioeconomic factors and other health disparities. Second, the study evaluated mother–infant bonding and risk for post-partum depression by validated screening tools [27–29]. The study included a diverse cohort of women with varying gravidity statuses to ensure its findings could be as broadly generalized as possible. It would be interesting to examine whether our results vary across different parity statuses, particularly among primiparous women. To account for this, we included parity in the multivariable model. Finally, possible confounding variables were controlled for in the comparison of delivery of a male vs a female infant, with similar maternal medical and obstetrical characteristics, including a direct assessment of maternal risk for depression.

Our study, being a cross-sectional study, is limited to assuming associations but not causation. Moreover, selection bias may occur, as women who suffer from present psychotic symptoms, suicidality, or substance abuse did not participate in our study. Moreover, the more severely depressed women might not have volunteered to participate. The self-report questionnaires evaluated mother–infant bonding at a single time point; therefore, a longitudinal pattern of impaired bonding was not obtained.

## Conclusion

We found better mother–infant bonding among Bedouin women delivering a male infant compared to a female infant. Our findings illustrate the value of the delivery of a male infant in the tribal patriarchal society of the Arab Bedouins, a minority group in Israel. Our study shows the significance of continuity of the family and emphasizes the cultural differences and the ethnic inequalities between the two study populations. Fetal gender and son preference is meaningful to many cultures and ethnic disparities, even today when modernization is in process and different styles of living occur. Feminist women challenge patriarchy by recreating a new model for women who retain tradition, but also adjust it to modern times. Further studies should be made to evaluate the gender preferences within the Bedouin society, comparing Bedouins who live in organized villages to those who live in rural unrecognized villages, to estimate the place of the modernization in the gender preferences. Attempts must be made to strengthen mother–infant bonding of the female gender and prevent impaired bonding from early conception.

## Data Availability

No datasets were generated or analysed during the current study.
